# Hypoparathyroidism: a brief historical overview for clinicians

**DOI:** 10.3389/fendo.2026.1769262

**Published:** 2026-04-01

**Authors:** Juan J. Díez

**Affiliations:** 1 Department of Endocrinology, Hospital Universitario Puerta de Hierro Majadahonda, Majadahonda, Spain; 2 Instituto de Investigación Sanitaria Puerta de Hierro Segovia de Arana, Majadahonda, Spain; 3 Department of Medicine, Universidad Autónoma de Madrid, Madrid, Spain

**Keywords:** calcium, history, hypoparathyroidism, parathyroid hormone, phosphorus

## Abstract

Hypoparathyroidism is a rare endocrine disorder characterized by deficient secretion of parathyroid hormone (PTH), resulting in hypocalcemia, hyperphosphatemia, and impaired mineral homeostasis. Although most cases are postsurgical, the disease encompasses a heterogeneous group of etiologies, including genetic, autoimmune, and infiltrative causes. For much of its history, hypoparathyroidism was considered unique among endocrine deficiencies in that it was not treated with hormone replacement, owing to limited understanding of parathyroid physiology and the absence of safe and effective PTH-based therapies. This review provides a historical perspective on the evolution of hypoparathyroidism, from the anatomical discovery of the parathyroid glands in the nineteenth century to recent advances in molecular biology, laboratory diagnostics, and targeted treatments. Key milestones include the elucidation of calcium and phosphate regulation, the isolation and characterization of PTH, the identification of the calcium-sensing receptor, and the discovery of regulatory pathways involving fibroblast growth factor 23 and klotho. Parallel advances in clinical chemistry enabled increasingly accurate measurement of serum calcium and PTH, facilitating improved diagnosis and disease monitoring. Therapeutic strategies have evolved from conventional treatment with calcium and active vitamin D toward physiological hormone replacement. Clinical development of recombinant PTH formulations, long-acting prodrugs, and novel receptor agonists has transformed the therapeutic landscape and renewed interest in disease-modifying approaches. Emerging therapies, including oral agents, long-acting formulations, and cell-based strategies, suggest that the management of hypoparathyroidism is entering a new era focused on restoring physiological mineral metabolism and improving long-term outcomes.

## Introduction

Hypoparathyroidism is an endocrine disorder in which insufficient production of parathyroid hormone (PTH) leads to disturbances in mineral metabolism, such as hypocalcemia and hyperphosphatemia (1, 2). Although multiple etiologies of impaired PTH production have been described, in approximately 75% of cases the condition results from inadvertent removal of or damage to the parathyroid glands during cervical surgery (postsurgical hypoparathyroidism). Other causes include autoimmune disorders, genetic syndromes, infiltrative diseases, medications, and, more rarely, glandular injury due to radiation or metastatic disease (3–6). The prevalence of this hormonal deficiency ranges from 6.4 to 38 cases per 100,000 inhabitants, according to studies conducted in different countries (3).

**Figure 1:**
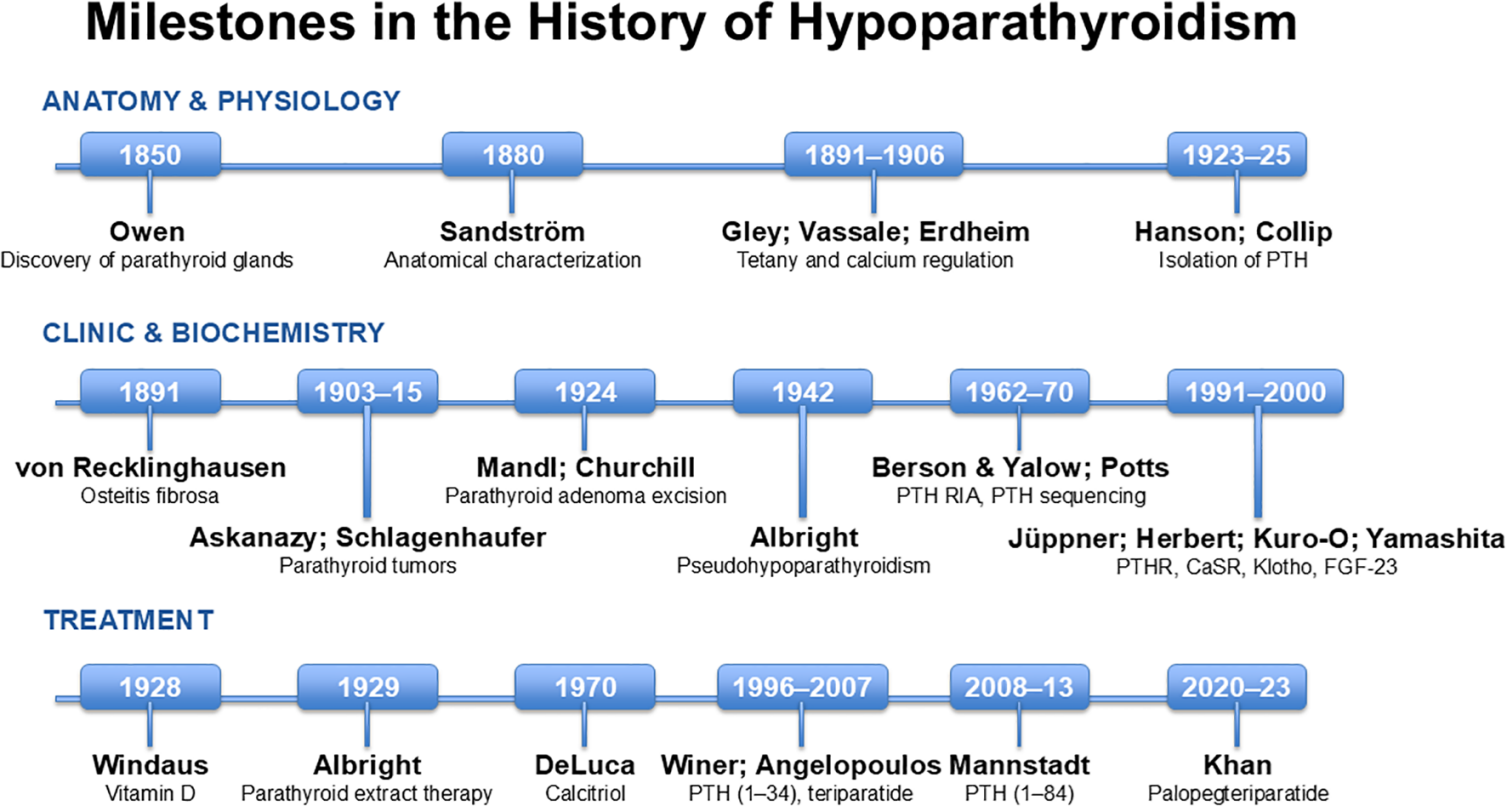
Historical overview of the most relevant milestones in the history of hypoparathyroidism, from the discovery of the parathyroid glands to the present day, classified into three distinct areas (anatomy and physiology, clinical and biochemical aspects, and treatment). PTH, parathyroid hormone; RIA, radioimmunoassay; PTHR, PTH receptor; CaSR, calcium-sensitive receptor; FGF-23, fibroblast growth factor 23.

Traditionally, medical and endocrinology textbooks have stated that hypoparathyroidism was the only hormonal deficiency not treated with replacement of the deficient hormone. This paradigm has persisted until very recently and is closely related to historical challenges in understanding the physiology and pathophysiology of these small glands, as well as to the difficulties in developing hormone replacement therapies with sufficient efficacy and safety for routine clinical use. The history of hypoparathyroidism spans the last two centuries and has evolved in parallel with the discovery of the parathyroid glands, the elucidation of their anatomy and physiology, and advances in the isolation and characterization of PTH. The following sections review some of the most significant milestones in the understanding of this rare disease from the nineteenth century to the present day (**Figure 1**).

## From discovery to anatomy

The first description of the parathyroid glands was provided by Sir Richard Owen in 1849, following an autopsy of an Indian rhinoceros (*Rhinoceros unicornis*) at the London Zoo. He noted “a small, compact, yellow glandular body attached to the thyroid at the point where the veins emerge” (7). Owen presented his observations to the Zoological Society in 1850 but did not assign any function to the newly described gland (8).

A decisive step came from Ivar Viktor Sandström, who described the anatomy and histology of parathyroid glands in dogs and humans at Uppsala University (9). He reported small, well-vascularized structures distinct from thyroid tissue, typically four glands—two on each side—embedded in the perithyroidal connective tissue. Based on their location, he termed them *glandulae parathyroideae*. Although his initial German manuscript was rejected for excessive length (10), he published the work in 1880 in *Upsala Läkareförenings Förhandlingar* (11) and presented the findings to the Stockholm Society of Natural Sciences (9, 10, 12). Sandström is therefore credited with the first detailed description of the parathyroid glands in humans and with coining the name that remains in use today.

## From anatomy to physiology

### Gley, Vassale, Erdheim

Modern thyroid surgery developed during the second half of the nineteenth century through the work of surgeons such as Billroth, Kocher, and Halsted. One of the major problems associated with surgical intervention on the thyroid gland was that accidental removal of the small parathyroid glands frequently resulted in tetany in affected patients, although the pathophysiological mechanisms linking cervical surgery to tetany were not yet understood. Eugène Gley first reported that accidental injury to parathyroid tissue during thyroidectomy in dogs led to tetany and death, suggesting a functional role for the glands, albeit without elucidating the mechanism (13). Giulio Vassale, with Francesco Generali, then showed that parathyroidectomy with an intact thyroid was sufficient to cause fatal paralytic and convulsive symptoms in animals (14). In parallel, Jakob Erdheim confirmed that animals with tetany lacked residual parathyroid tissue.

Subsequent studies demonstrated that parathyroid autotransplantation prevented post-parathyroidectomy neuromuscular symptoms (15, 16). At that time, a prevailing hypothesis proposed that the glands secreted a detoxifying factor that removed toxins responsible for tetany (17). A particularly intriguing observation was that dogs subjected to total thyroidectomy and fed meat but not milk, both before and after surgery, continued to experience tetanic convulsions. This finding suggested that calcium contained in milk might exert a protective effect against tetany.

### MacCallum, Voegtlin

William G. MacCallum proposed that tetany was a direct consequence of calcium deficiency, demonstrating that dialysis-induced hypocalcemia in dogs produced tetany and inferring that post-parathyroidectomy tetany arose from calcium depletion (18). In 1909, together with Carl Voegtlin, he argued that “apparently the parathyroids control calcium, so that after their removal a rapid excretion, possibly accompanied by insufficient absorption, deprives the tissues of calcium.” The reasoning included an analogy between destruction of the pancreatic islets of Langerhans, which results in altered carbohydrate metabolism, and excision of the parathyroid glands, which leads to abnormal calcium excretion and symptoms of tetany—thus coining the term “calcium diabetes” (*diabetes calcaricus*) (19).

### Hanson, Collip

In 1924, Adolph Hanson first reported isolation of an active substance from bovine parathyroid glands (20). James B. Collip subsequently isolated a biologically active parathyroid extract by homogenizing bovine glands, acid extraction, concentration, and neutralization, thereby demonstrating the role of parathyroid hormone (PTH) in calcium regulation and resolving long-standing debates about parathyroid function (21). This breakthrough resolved longstanding debates regarding parathyroid function and marked the beginning of a new era in the understanding of calcium metabolism and the clinical management of parathyroid disorders, representing a landmark achievement in the history of endocrinology (22, 23).

Many years later, Howard Rasmussen and Lyman C. Craig succeeded in purifying the hormone (24). In 1952, they demonstrated that countercurrent distribution techniques allowed the production of PTH preparations that were significantly purer than those obtained using previously available methods. This advance enabled the isolation of hormone fractions with greater biological activity and reduced contamination by nonspecific proteins, thereby improving biochemical characterization of the hormone.

## From physiology to clinical practice

In the nineteenth century, Chvostek and Trousseau described the classical clinical signs of hypocalcemic tetany, although their underlying pathophysiological basis was not yet known. Several years passed before the relationship between the parathyroid glands, calcium and phosphate homeostasis, and bone metabolism was fully understood.

### Von Recklinghausen, Askanazy, Erdheim, Schlagenhaufer, Mandl

The association between parathyroid glands and bone disease was not immediately recognized. In 1891, the German pathologist Friedrich Daniel von Recklinghausen contributed a chapter entitled *“Die fibröse oder deformirende Ostitis, die Osteomalacie und die osteoplastische Carcinose in ihren gegenseitigen Beziehungen”* (“Fibrous or deforming osteitis, osteomalacia, and osteoplastic carcinomatosis in their mutual relationships”) to a volume commemorating the seventy-first anniversary of Rudolf Ludwig Virchow. In this chapter, he described what is now known as osteitis fibrosa cystica, although he did not relate the condition to the parathyroid glands. The disease had previously been described by Eberhard Engel, but neither author attributed the skeletal changes to parathyroid dysfunction (25).

Max Askanazy later described a woman with fibrous osteitis and a 4.5-cm parathyroid tumor. However, he did not consider hypertrophy of this single gland to be relevant to the bone pathology (26). Erdheim, for his part, observed parathyroid hyperplasia in three women with osteomalacia and assumed that parathyroid changes were secondary to bone disease. In contrast, in 1915, Friedrich Schlagenhaufer proposed a different interpretation. He noted that some patients with osteitis fibrosa cystica had only one enlarged parathyroid gland and suggested that parathyroid abnormalities were the cause, rather than the consequence, of bone disease (27). He was the first to propose excision of the enlarged parathyroid gland as a potential treatment for the skeletal disorder.

The first successful parathyroidectomy was not performed until 1924. Felix Mandl, a Viennese surgeon, treated Albert Ghane [Jhane], a 34-year-old tram driver with clinical and radiological features of osteitis fibrosa cystica. In accordance with prevailing theories regarding the detoxifying role of the parathyroid glands, Mandl initially treated the patient with Collip’s extract. When no improvement was observed, he attempted transplantation of four parathyroid glands from a deceased accident victim, again without success (28). Finally, in 1925, accepting Schlagenhaufer’s hypothesis, Mandl performed cervical exploration and excised a tumor in the lower left thyroid region near the recurrent laryngeal nerve, identifying three apparently normal parathyroid glands (29). Histological examination, reported among others by Erdheim, confirmed the lesion as a parathyroid adenoma.

Captain Charles Martell was the first patient with primary hyperparathyroidism to undergo surgery in the United States. He suffered from bone pain, muscle weakness, loss of height, and pectus carinatum, as well as osteomalacia and fractures. Laboratory findings revealed hypercalcemia, hypophosphatemia, and hypercalciuria—biochemical abnormalities similar to those observed with excessive administration of Collip’s extract. He underwent several unsuccessful surgical procedures performed by leading surgeons of the time. Ultimately, Edward Churchill excised an adenoma located in the superior mediastinum. Notably, the patient developed postoperative tetany, which was treated with Collip’s extract and intravenous calcium. He eventually died from laryngospasm, a well-known complication of hypocalcemia due to hypoparathyroidism (30–32).

### Beumer, Falkenheim, Albright

From early 20th-century reports of postoperative tetany to contemporary endocrine surgery, postsurgical hypoparathyroidism emerged as the predominant adult phenotype, typically following thyroid or other anterior neck operations that injure or devascularize the parathyroid glands. Advances in endocrine surgery—routine gland identification and preservation, meticulous hemostasis, and selective parathyroid autotransplantation—progressively lowered the burden of long-term sequelae and sharpened the clinical distinction between transient and persistent courses. In parallel, the non-surgical spectrum—less common but etiologically heterogeneous—coalesced around autoimmune forms and genetic syndromes or variants, as well as infiltrative and magnesium-related causes, a taxonomy that crystallized with clinical genetics and updated international guidance (33–35).

The first clinical case of non-surgical hypoparathyroidism was described by Hans Beumer and Curt Hermann Falkenheim in 1926. They reported a patient without skeletal abnormalities who presented with chronic tetany, muscle spasms, carpopedal cramps, and seizures, whose biochemical studies revealed profoundly low serum calcium levels accompanied by elevated serum phosphate concentrations. They coined the term *idiopathische Tetanie* and proposed the existence of a hormonal deficiency as the cause of the disorder (36).

Fuller Albright is regarded as one of the most influential endocrinologists of the twentieth century due to his seminal contributions to the study of calcium metabolism and parathyroid gland function. He conducted his entire professional career at Massachusetts General Hospital, where he founded one of the first clinical research units in endocrinology and continued his work until his death from a neurological disease. At the age of 29, he clearly defined the characteristic biochemical profile of hypoparathyroidism and demonstrated that parathyroid gland extracts could alleviate the symptoms of tetany, thereby establishing foundational principles of the endocrinology of bone and mineral metabolism (37, 38). In subsequent decades, additional cases of hypoparathyroidism were reported. Notably, the pediatrician Lachman compiled approximately 70 cases between 1926 and 1939, and Dietrich published 113 cases identified in the literature up to 1952 (39).

In 1942, Fuller Albright also identified the first cases of pseudohypoparathyroidism. This syndrome was characterized by hypocalcemia, hyperphosphatemia, and distinctive phenotypic features—including short stature, round facies, brachydactyly, obesity, frontal hyperostosis, and intracranial calcifications—now collectively referred to as Albright hereditary osteodystrophy. Several forms of pseudohypoparathyroidism have since been described, all characterized by elevated PTH concentrations due to peripheral resistance to the hormone, despite differing phenotypes (37, 38).

### Thorpe, Handley, Whitaker, Neufeld

The earliest descriptions of associations between autoimmune endocrine diseases, including hypoparathyroidism, date back to the initial work of Edward S. Thorpe and Harry E. Handley in 1929 (40), as well as Whitaker in 1943 (41, 42). However, a clear definition and classification of these syndromes were provided by Michel Neufeld, Noel MacLaren, and Robert Blizzard in 1972. In an extensive review of 295 patients with Addison’s disease associated with other autoimmune conditions, they established distinctions among the different autoimmune polyglandular syndromes. In doing so, they clearly delineated the association of Addison’s disease with mucocutaneous candidiasis and acquired hypoparathyroidism, now known as autoimmune polyglandular syndrome type I (43, 44).

## From clinical practice to molecular biology

### Potts, Niall

The purification of PTH in the 1950s represented a crucial step toward subsequent sequencing and structural analyses, enabling the development of more precise approaches to the study of parathyroid physiology and the diagnosis of disorders related to calcium metabolism. As mentioned above, Rasmussen and Craig observed that the hot acid extraction method caused fragmentation of the peptide and therefore opted for extraction using organic solvents. This approach successfully achieved the desired goal: releasing the active principle—the parathyroid polypeptide—separated from other cellular components without fragmentation. These studies enabled the sequencing and synthesis of both bovine PTH (45–47) and human PTH (48–51).

Particularly noteworthy are the studies conducted by the group led by John T. Potts and Harold D. Niall. In 1970, these investigators reported the complete 84–amino acid sequence of bovine PTH (45). Several years later, they published the amino-terminal sequence of human PTH (48, 52). These investigations are now regarded as foundational, as they laid the groundwork for the subsequent development of specific immunoassays and for the therapeutic use of PTH-derived peptides based on biologically active hormone fragments (53).

### Jüppner

During the final decade of the twentieth century, many aspects of calcium physiology and the regulation of bone metabolism by PTH were clarified through several landmark discoveries. The identification of the primary structure and cloning of the parathyroid hormone receptor are credited to Harald Jüppner and colleagues. In an elegant study published in *Science*, the authors described and cloned complementary DNA encoding a 585–amino acid receptor with seven transmembrane domains. The expressed receptor bound both PTH (type 1 PTH receptor, PTH1R) and parathyroid hormone–related peptide (PTHrP) with equal affinity, and both ligands stimulated adenylate cyclase to a similar extent. The authors noted that the striking homology with the calcitonin receptor, together with the lack of homology with other G protein–coupled receptors, indicated that receptors for calcium-regulating hormones were related and constituted a novel receptor family (54). Subsequent high-resolution studies further elucidated the interactions between PTH and its receptor, including crystallographic and cryo-electron microscopy analyses, allowing visualization of the molecular details of receptor binding and activation (55–58).

### Herbert, Brown

The inverse relationship between serum ionized calcium concentration and PTH secretion had been recognized since the introduction of radioimmunoassays for PTH quantification in the 1960s (59). Experiments conducted in the 1980s demonstrated that extracellular calcium, even in the presence of calcium channel blockers, exerted a depolarizing effect on parathyroid cells, mirroring the inverse relationship between calcium levels and PTH secretion (60). Subsequent studies revealed that increases in extracellular calcium stimulated the production of inositol 1,4,5-trisphosphate and diacylglycerol, second messengers known to be associated with receptor-mediated intracellular calcium mobilization (61, 62).

Further investigations by Edward F. Nemeth and Antonio Scarpa demonstrated that extracellular divalent cations induced rapid increases in intracellular calcium mobilization in parathyroid cells in the absence of calcium influx across the plasma membrane (63). Collectively, these findings pointed to the existence of a membrane receptor in parathyroid cells capable of sensing extracellular calcium concentrations and regulating PTH secretion via intracellular calcium mobilization (64).

Finally, in 1993, the calcium-sensing receptor (CaSR) was discovered and cloned by the group led by Steven C. Herbert and Edward M. Brown (65). These investigators isolated and characterized the receptor from bovine parathyroid tissue, identifying CaSR as a class C G protein–coupled receptor that plays a fundamental role in the regulation of extracellular calcium homeostasis (66, 67). Identification of this receptor not only improved understanding of the mechanisms governing serum calcium regulation but also enabled the development of novel therapeutic agents, such as calcimimetics and calcilytics, which are currently used in the treatment of parathyroid disorders (68).

### Yamashita

In the present century, additional pivotal discoveries have advanced understanding of phosphate–calcium metabolism, including the identification of the phosphaturic hormone fibroblast growth factor 23 (FGF-23) and the protein klotho (69, 70). FGF-23 was first identified in 2000 by the group led by Tetsuo Yamashita. These investigators isolated murine complementary DNA encoding a novel fibroblast growth factor consisting of 251 amino acids, which they named FGF-23 as the twenty-third documented member of the FGF family. They also isolated the human complementary DNA encoding FGF-23 (251 amino acids), which shares approximately 72% amino acid homology with the murine protein and exhibits similarity to other FGFs such as FGF-21 and FGF-19 (71). Currently, therapies that neutralize excess FGF23—such as burosumab—are indicated for FGF23-mediated hypophosphatemic disorders (e.g., X-linked hypophosphatemia and tumor-induced osteomalacia) and are not used to treat hypoparathyroidism (72, 73).

Subsequent experimental and clinical studies by investigators including Beate Lanske, Marie Courbebaisse, Kenneth E. White, R. G. Erben, and Clemens Bergwitz clarified the effects of this novel hormone on phosphate regulation, vitamin D metabolism, and its interaction with PTH. These studies demonstrated that FGF-23 inhibits tubular phosphate reabsorption by downregulating expression of the NaPi-2a and NaPi-2c cotransporters in the renal proximal tubule and reduces renal synthesis of calcitriol, thereby decreasing intestinal absorption of calcium and phosphate (74, 75). Although its most prominent physiological effect is increased phosphaturia through mechanisms distinct from those of PTH, FGF-23 was also shown to inhibit PTH secretion (74, 76). It is now known that FGF-23 acts directly on the parathyroid gland via the klotho–FGFR receptor complex, reducing PTH gene expression and secretion through activation of the MAPK signaling pathway (72–80). This inhibitory effect has been demonstrated both *in vivo* and *in vitro* in animal models and cell cultures (76, 81).

These findings have clinical relevance. Although FGF-23 biology is not the primary driver of hypoparathyroidism, circulating FGF-23 concentrations are frequently elevated in chronic PTH deficiency, reflecting the combined effects of persistent hyperphosphatemia and reduced renal calcitriol synthesis—two well−established stimuli for FGF23 secretion (82). However, despite elevated FGF-23 levels, phosphate excretion remains inadequate, and most patients continue to exhibit hyperphosphatemia, indicating a state of relative renal resistance to FGF-23. Mechanistically, the absence of PTH reduces expression of the NaPi−2a and NaPi−2c cotransporters to a lesser degree than FGF-23 alone, and the phosphaturic actions of FGF-23 appear insufficient to compensate for the lack of PTH−mediated downregulation (83). Moreover, impaired 1α−hydroxylase activity in hypoparathyroidism leads to lower calcitriol levels, which may further blunt FGF-23 signaling in the proximal tubule (84). As a result, FGF-23 elevation becomes physiologically ineffective, contributing to the characteristic combination of high serum phosphate, low or inappropriately normal calcitriol, and an increased calcium–phosphate product. These alterations underscore that, despite its mechanistic links to phosphate homeostasis, FGF23−targeted therapies have no therapeutic role in hypoparathyroidism, as the predominant defect remains PTH deficiency rather than FGF23 excess.

### Kuro-O

Makoto Kuro-O and colleagues discovered klotho protein in 1997. They identified the *klotho* gene as a suppressor of aging in a murine model of premature aging associated with disturbances in mineral metabolism (85–89). It was subsequently demonstrated that the transmembrane form of klotho functions as an essential co-receptor for FGF-23 signaling, enabling its physiological actions on the kidney and other organs (69). Kuro-O named the protein after Clotho, one of the three Moirai of Greek mythology, symbolizing longevity and the thread of life, as she was responsible for spinning the thread of human fate (86, 90). These pathophysiological considerations underscore that, while FGF23/klotho biology interfaces with PTH signaling in the parathyroid, anti-FGF23 therapy addresses hypophosphatemia due to FGF23 excess, rather than PTH deficiency (76).

## From molecular biology to the clinical laboratory

### Calcium

Otto Folin, a chemist at Harvard University renowned for his work on protein-free blood analysis methods, was the first to measure serum calcium in 1904. He employed a colorimetric method based on formation of a colored complex between calcium and oxalate, followed by titration of the precipitated calcium oxalate (91). Following these early analyses, more specific colorimetric methods were developed and became standard in clinical laboratories due to their good correlation with reference methods and low interference from hemolysis, lipemia, or icterus. More recently, enzymatic methods and alternative chelating agents have been introduced, offering improved linearity, reagent stability, and analytical precision (92, 93). Currently, atomic absorption spectrometry and isotope-dilution mass spectrometry allow highly precise and accurate quantification of serum calcium with excellent reproducibility (94–96).

### PTH

Accurate diagnosis of parathyroid disorders required reliable methods for determining serum PTH concentrations. Although experimental bioassays in animals were performed during the 1920s and 1930s, a major breakthrough occurred in 1963 when Solomon A. Berson and Rosalyn Yalow developed the first radioimmunoassay for PTH (97). These investigators used a unique polyclonal antibody raised against bovine PTH and labeled with iodine-125, enabling, for the first time, quantification of circulating PTH in serum. The method required separation of antibody-bound hormone from the free fraction, typically through solid-phase immunoprecipitation. However, significant limitations existed, including low sensitivity and limited specificity due to antibodies directed against mid-region or C-terminal epitopes, poor discrimination of bioactive forms, and interference from PTH fragments (98–102). The pioneering work of these investigators was recognized with the Nobel Prize awarded to Rosalyn Yalow.

Following these first-generation assays, second-generation “sandwich” immunoassays were developed in the 1980s. These assays employed two antibodies directed against the N-terminal and C-terminal regions of PTH, allowing capture of the intact 84–amino acid molecule. Nevertheless, they also exhibited cross-reactivity with truncated PTH fragments, detecting not only PTH 1–84 but also inactive forms. This limitation was particularly problematic in patients with renal failure, in whom such fragments accumulate and may lead to overestimation of biologically active PTH (98, 99).

Current third-generation assays aim to measure exclusively the intact PTH molecule (PTH 1–84), excluding truncated fragments and thereby improving specificity. However, issues related to assay standardization and inter-method variability persist (101, 103). More recently, liquid chromatography coupled with tandem mass spectrometry (LC-MS/MS) has been proposed as a reference method, as it enables precise and simultaneous quantification of PTH 1–84 and its fragments using international calibration standards. This approach facilitates harmonization of results and more accurate definition of reference intervals (103–105).

These advances in clinical laboratory methods have been translated into clinical practice and the correct classification of patients. The availability of early postoperative PTH assays and harmonized biochemical criteria enabled more precise definition of different types of postsurgical hypoparathyroidism. Historically, clinicians noted that many patients developed transient postoperative hypocalcemia with recovery within weeks to months, whereas a subset progressed to persistent deficiency. Nowadays, current international statements consider postsurgical hypoparathyroidism “permanent” when persisting beyond 12 months, while also recognizing late remissions, underscoring the need for longitudinal reassessment (6, 106–108).

## From the laboratory to treatment

### Pathophysiological consequences of PTH deficiency

**Figure 2:**
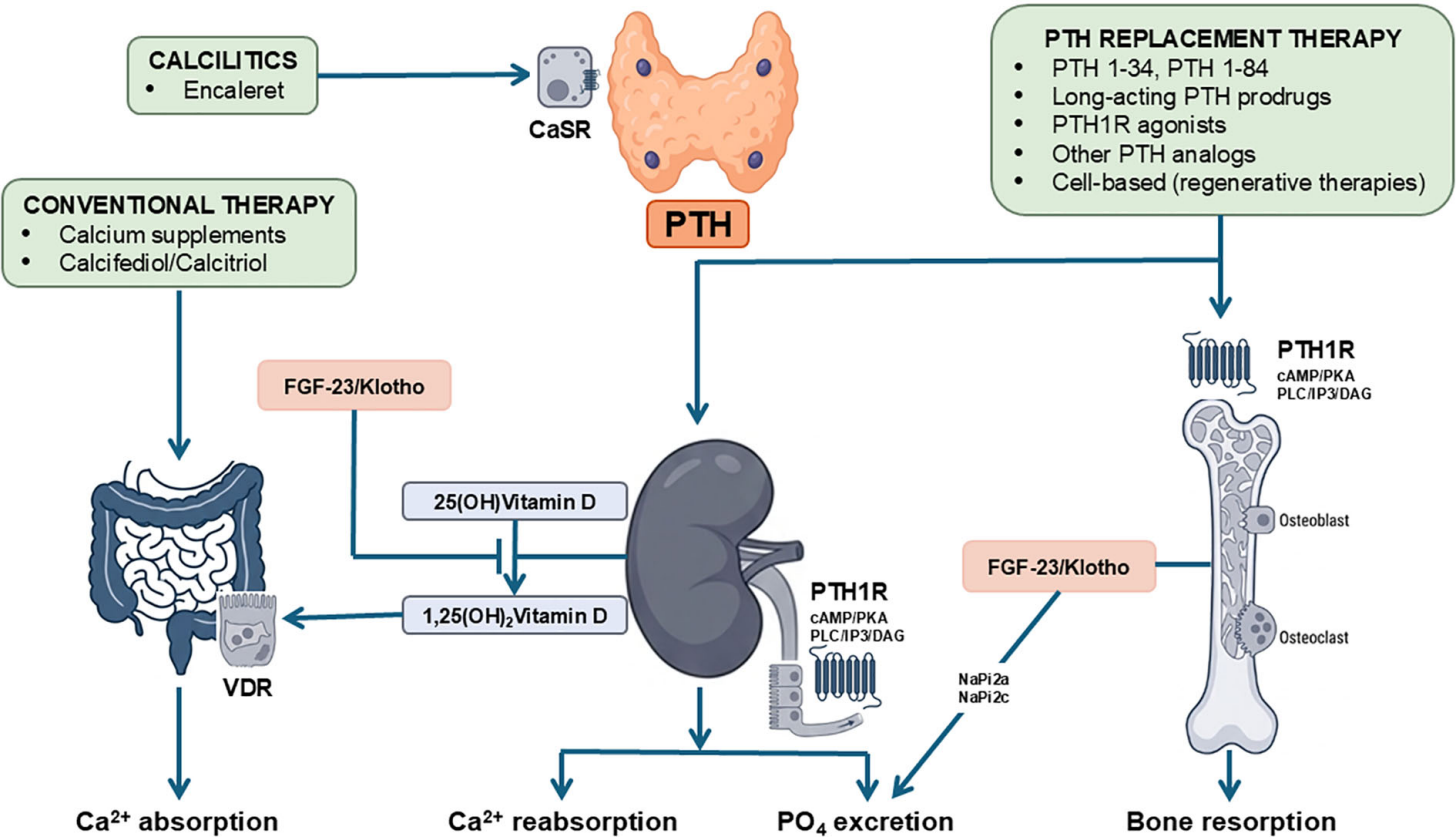
Mechanistic overview of PTH signaling pathways and therapeutic targets in hypoparathyroidism. The figure summarizes canonical parathyroid hormone (PTH) signaling and illustrates how current and emerging therapies act along this axis. Binding of PTH to the type-1 PTH receptor (PTH1R) activates Gs–adenylate cyclase–cAMP–protein kinase A and Gq–phospholipase C–IP3/DAG pathways, coordinating effects across bone, kidney, and intestine. In bone, PTH modulates remodeling dynamics and context-dependent calcium mobilization; in the kidney, it increases distal tubular calcium reabsorption, downregulates proximal NaPi-2a/2c transporters to promote phosphaturia, and stimulates 1α-hydroxylase activity to raise 1,25(OH)_2_ vitamin D (calcitriol); in the intestine, calcitriol enhances active calcium absorption. Therapeutic classes are mapped to their primary points of action: conventional therapy (oral calcium plus active vitamin D/calcifediol) augments intestinal calcium flux but does not restore renal PTH-dependent handling; PTH-based replacement (PTH 1–34, PTH 1–84, long-acting PTH prodrugs, PTH1R agonists, and other analogs) re-establishes physiological signaling at the receptor level, reducing supplement burden and often lowering urinary calcium; CaSR-targeted therapies (calcilytics, e.g., encaleret) act upstream by attenuating gain-of-function *CASR* activity to normalize PTH secretion in autosomal dominant hypocalcemia type 1, but are not used for classical hypoparathyroidism. The FGF-23/klotho axis is depicted to contextualize phosphate regulation and its crosstalk with PTH and vitamin D metabolism; while FGF-23 is frequently elevated in chronic hypoparathyroidism, its phosphaturic effect is often insufficient in the absence of PTH-mediated proximal tubular regulation. cAMP, cyclic adenosine monophosphate; CaSR, calcium-sensing receptor (protein); DAG, diacylglycerol; FGF-23, fibroblast growth factor 23; IP3, inositol 1,4,5-trisphosphate; NaPi-2a/2c, sodium–phosphate cotransporters type 2a/2c; PKA, protein-kinase A; PLC, phospholipase C; PTH, parathyroid hormone; PTH1R, type-1 PTH receptor; VDR, vitamin D receptor; 25(OH) vitamin D, 25-hydroxyvitamin D (calcifediol); 1,25(OH)_2_ vitamin D, 1,25-dihydroxyvitamin D (calcitriol).

PTH maintains extracellular calcium and phosphate homeostasis by coordinating bone remodeling, renal tubular transport, and intestinal mineral absorption (via vitamin D activation). A schematic representation of the PTH signaling pathways in the different organs appears in **Figure 2**.

In chronic hypoparathyroidism, the absence or insufficiency of PTH signaling produces a characteristic biochemical triad—hypocalcemia, hyperphosphatemia, and (in many patients) hypercalciuria—that underlies the cardinal clinical manifestations and informs therapeutic choices (1–4). In fact, PTH is a principal regulator of renal calcium reabsorption (predominantly in the distal nephron), phosphate excretion (proximal tubule NaPi-2a/2c downregulation), and 1α-hydroxylase activity (CYP27B1) driving calcitriol synthesis. When PTH is deficient, distal tubular calcium reabsorption falls, so even when serum calcium is normalized with supplements, urinary calcium excretion tends to be inappropriately high (relative to serum calcium), predisposing to nephrolithiasis and nephrocalcinosis (1, 109). Concomitantly, loss of PTH-mediated phosphaturia increases tubular phosphate reabsorption, producing hyperphosphatemia; this, together with low/normal calcitriol, raises the calcium–phosphate product and risk of extraskeletal calcification. Finally, reduced renal 1α-hydroxylase activity lowers endogenous calcitriol generation, impairing intestinal calcium absorption and perpetuating hypocalcemia (109, 110).

On the skeleton, PTH exerts dual, context-dependent actions: intermittent exposure favors anabolic effects, whereas continuous elevation increases bone remodeling with net resorption. In PTH deficiency, the remodeling rate is suppressed, osteoclastic activity is reduced, and bone turnover markers decline. Histomorphometric and densitometric studies show increased bone mineral density at trabecular-rich sites, thickened trabeculae, and reduced activation frequency; however, higher BMD does not necessarily translate into proportionate mechanical benefit, and skeletal dynamics differ from both euparathyroid states and hyperparathyroid bone disease (111, 112). Clinically, reduced skeletal calcium mobilization limits the capacity to buffer fluctuations in serum calcium, contributing to symptomatic hypocalcemia and cramps/tetany in decompensated states (113).

In addition to the above actions, calcitriol—whose renal synthesis is stimulated by PTH—upregulates TRPV6, calbindin, and other epithelial transport machinery to enhance active intestinal calcium absorption. In hypoparathyroidism, endogenous calcitriol levels are reduced (or inappropriately normal), leading to impaired intestinal calcium uptake and contributing to hypocalcemia despite adequate dietary intake (114). Pharmacologic calcitriol or alfacalcidol compensates for this defect by restoring intestinal absorption, but does not correct the renal-tubular handling abnormalities driven by PTH absence (115).

In summary, the convergence of diminished intestinal calcium absorption (low calcitriol), reduced distal tubular calcium reabsorption (absent PTH), and enhanced phosphate retention (absent PTH-mediated phosphaturia) explains the hallmark triad of hypocalcemia, hyperphosphatemia, and hypercalciuria in treated patients. Conventional therapy with oral calcium plus active vitamin D corrects hypocalcemia primarily by increasing intestinal calcium flux, but leaves the renal-tubular PTH deficit unaddressed, so hypercalciuria may persist and require careful titration and thiazide adjuncts (115, 116). By contrast, physiological replacement using various PTH preparations (see below) more directly restores distal tubular calcium reabsorption, re-establishes phosphaturiaphosphaturia, and normalizes endogenous calcitriol generation, thereby lowering calcium/active vitamin D requirements and often reducing urinary calcium (**Figure 2**).

### Calcium and active vitamin D (conventional therapy)

For more than a century—and still today—oral calcium salts together with active vitamin D analogues have constituted the therapeutic backbone for chronic hypoparathyroidism. The paradigm originates from early clinical observations: in 1909, MacCallum and Voegtlin reported that calcium administration alleviated postsurgical tetany, anchoring the concept that hypocalcemia drives neuromuscular excitability (19). Subsequent advances in vitamin D chemistry provided the necessary tools to sustain serum calcium: Adolf Windaus elucidated the structures of ergocalciferol (vitamin D_2_) and cholecalciferol (vitamin D_3_) (117, 118), and later Hector F. DeLuca characterized the active metabolites—25-hydroxyvitamin D and 1,25-dihydroxyvitamin D (calcitriol)—while mapping the hepatic and renal hydroxylations that underlie vitamin D activation (117–121). These contributions established the endocrine framework supporting routine clinical use of vitamin D metabolites and calcium in hypoparathyroidism (122, 123). In clinical terms, this supplementation strategy compensates for absent PTH by enhancing intestinal calcium absorption and providing substrate, but it does not restore physiological, PTH-driven renal calcium reabsorption or dynamic phosphate regulation—limitations that motivated the development of hormone-replacement approaches (35).

In real-world practice, conventional therapy remains appropriate for many adults with stable biochemical control, manageable pill burden, and no clinically relevant hypercalciuria or renal complications. Targets include maintaining normocalcemia (or low-normal calcium), minimizing symptoms, and avoiding hypercalciuria while monitoring renal risk. These aims and monitoring practices align with current consensus frameworks that continue to position calcium plus active vitamin D as first-line therapy, with periodic reassessment of control and renal safety (6, 106).

Among available calcium supplements, calcium carbonate and calcium citrate are the most widely used in chronic hypoparathyroidism, and their differing pharmacological profiles have relevant clinical implications. Calcium carbonate contains the highest proportion of elemental calcium (~40%), is inexpensive, and is typically taken with meals because its absorption depends on gastric acidity; reduced acid secretion (e.g., in older adults, patients on proton-pump inhibitors, or those with achlorhydria) may impair its bioavailability. In contrast, calcium citrate contains ~21% elemental calcium but has superior absorption in achlorhydric states and allows greater dosing flexibility. It is also associated with less gastrointestinal discomfort, particularly less bloating and constipation, which are frequently reported with carbonate salts (1, 2, 124).

Despite these differences, both formulations can contribute to urinary calcium elevation, a central limitation of supplementation-based therapy in hypoparathyroidism. High doses are often required to maintain serum calcium in the target range, and the combination of increased intestinal calcium load and absent PTH-mediated distal tubular reabsorption predisposes patients to hypercalciuria, nephrolithiasis, and nephrocalcinosis. These considerations highlight why conventional therapy, although long-standing and effective for many patients, cannot fully replicate the physiological renal actions of PTH. Such limitations underpin the development of PTH-based replacement strategies, which more directly restore mineral homeostasis and reduce the need for high-dose calcium and active vitamin D (6, 35, 124, 125).

### PTH therapy (physiological hormone replacement)

Early bioactive parathyroid extracts demonstrated the canonical physiological actions of PTH—increasing serum calcium, raising urinary calcium, lowering serum phosphate, and increasing urinary phosphate—as reported by Albright and Ellsworth (126). Decades later, intermittent PTH 1–34 (teriparatide) was explored in hypoparathyroidism. In a 15-month study, Winer, Yanovski, and Cutler Jr. showed that PTH 1–34, combined with background calcitriol and calcium, maintained normocalcemia with lower urinary calcium excretion than calcitriol alone, albeit with increases in bone turnover markers (127). Although teriparatide was developed and approved for osteoporosis (128, 129) rather than hypoparathyroidism, off-label experience suggested benefit in selected patients with poor control or hypercalciuria on conventional therapy; initial case reports and subsequent series described improved serum calcium stability and reduced calciuria, with administration as intermittent injections or continuous subcutaneous infusion in challenging cases (130–137).

Between 2008 and 2013, clinical programs evaluated PTH 1–84, the full-length hormone (138, 139). In 2013, Mannstadt et al. demonstrated that subcutaneous PTH 1–84 enabled ≥50% reductions in daily calcium and active vitamin D while maintaining normocalcemia in a substantial proportion of patients (139). Regulatory milestones followed: in 2015, the FDA approved Natpara^®^ (PTH 1–84) for chronic hypoparathyroidism in adults, and in 2017 the EMA granted conditional approval; production was later discontinued by the manufacturer due to supply issues, which has influenced availability but not the underlying therapeutic rationale of physiological hormone replacement in selected patients.

In recent years there has been much debate about who should be candidates for PTH replacement therapy. Contemporary consensus documents and expert reviews converge on a similar clinical profile for escalation from supplementation to PTH: inadequate biochemical control (despite optimized calcium/active vitamin D), symptomatic fluctuations, persistent hypercalciuria or renal complications, intolerance or excessive pill burden, and/or impaired quality of life attributable to the disease or to conventional regimens. The therapeutic intent is to restore a more physiological mineral homeostasis, reduce supplementation needs, and improve renal safety and quality of life (6, 35).

The regulatory history of PTH-based therapies is closely linked to safety considerations, most notably the osteosarcoma signal observed in rats exposed to high lifetime doses of PTH analogues. Although this finding has not been replicated in humans, it contributed to the cautious regulatory approach applied to agents such as PTH 1–34 and PTH 1–84 (140, 141). Post-marketing surveillance and clinical trial data have primarily identified mild to moderate adverse events, including hypercalcemia, hypercalciuria, transient nausea, and injection-site reactions, with serious events being uncommon. Observational cohorts have not demonstrated an increased risk of malignancy or osteosarcoma in humans receiving therapeutic doses, though continued vigilance is recommended (138, 139).

### Long-acting PTH modalities and targeted agents

To approximate more stable, physiologic PTH exposure, palopegteriparatide (TransCon PTH) couples a PTH 1–34 payload to a transient carrier–linker system to enable once-daily release (142). In the PaTHway trial, 79% of treated adults achieved the primary endpoint of independence from conventional therapy while maintaining normocalcemia without up-titration during the final evaluation window (143), and the drug has recently obtained EMA approval for adults with hypoparathyroidism. Additional innovations include eneboparatide, a PTH1R agonist that, in early investigation, yielded >88% independence from conventional therapy (144), and encaleret, an oral CaSR antagonist (calcilytic) used in autosomal dominant hypocalcemia type 1 due to gain-of-function *CASR* mutations, aligning mechanism with genotype (145).

For these newer and long-acting formulations, safety profiles to date appear consistent with the class, but long-term renal outcomes, skeletal effects, and class-specific risks remain areas of active study given their recent introduction. These considerations underscore the importance of individualized treatment selection and continued post-approval monitoring as PTH-based replacement regimens evolve.

### Emerging therapies

Beyond these parenteral and receptor-targeted approaches, oral PTH formulations are being explored to deliver stabilized PTH analogues via permeation-enhanced platforms with the goal of reproducing physiologic exposure while avoiding injections (146–148). Although still investigational, these strategies aim to reduce pill burden and improve day-to-day biochemical stability (147). In parallel, microencapsulation of parathyroid cells within selectively permeable biomaterials (149, 150) seek to restore sustained endogenous PTH secretion. Other efforts include once-weekly PTH analogues, such as MBX-2109, designed to provide sustained receptor activation with lower injection frequency. Initial trial data suggest the potential for stable serum calcium control with reduced supplement requirements, although longer-term outcomes and renal safety remain under investigation (151). Together, these platforms illustrate how technological advances continue to broaden the therapeutic landscape and may offer future alternatives for patients who remain suboptimally controlled with existing approaches (**Table 1**).

**Table 1:** Summary of the evolution of current treatment studies and future perspectives in the therapy of hypoparathyroidism.

Year	Molecule	Key event	Main reference
1996	PTH 1-34	First use of PTH 1–34 in humans	Winer et al., 1996 (114)
1998-2010	PTH 1-34	Physiological and pediatric studies	Winer et al., 1998 (123); Winer et al., 2010 (124)
2007	Teriparatide	First off-label use in hypoparathyroidism	Angelopoulos et al., 2007 (117)
2010–2013	PTH 1-84	Clinical trials of efficacy and safety	Rubin et al., 2010 (125); Mannstadt et al., 2013 (126)
2019	Microencapsulation	Encapsulation of cells with a semipermeable coating for allotransplantation	Yucesan et al., 2019 (135)
2021	EBP02 (EnrteraBio)	Oral formulation of PTH 1-34	Ish-Shalom et al., 2021 (131)
2020–2023	TransCon PTH	Development of TransCon PTH (palopegteriparatide) in humans	Karpf et al., 2020 (127); Khan et al., 2023 (128)
2024	Eneboparatide	Development of a PTH receptor agonist in humans	Tackacs et al., 2024 (129)
2024	EB612 (EnteraBio)	Oral PTH 1–34 analog	Burshtein et al., 2024 (132)
2024	SP-1462 (Septerna, Inc.)	Small molecule oral PTHR1 agonist	Zhang et al., 2024 (133)
2025	MBX 2109 (MBX Biosciences)	Prodrug (acylated PTH)	Carney et al., 2025 (136)

Finally, we can also mention some additional emerging platforms. These are several innovative modalities that aim to further approximate physiological exposure to PTH. One investigational strategy is mRNA-based PTH replacement, exemplified by the therapeutic candidate XH02, which delivers a stabilized mRNA transcript encoding full-length PTH to promote endogenous production. Early-phase clinical evaluation is ongoing (NCT07197450) (152, 153).

**Figure 3:**
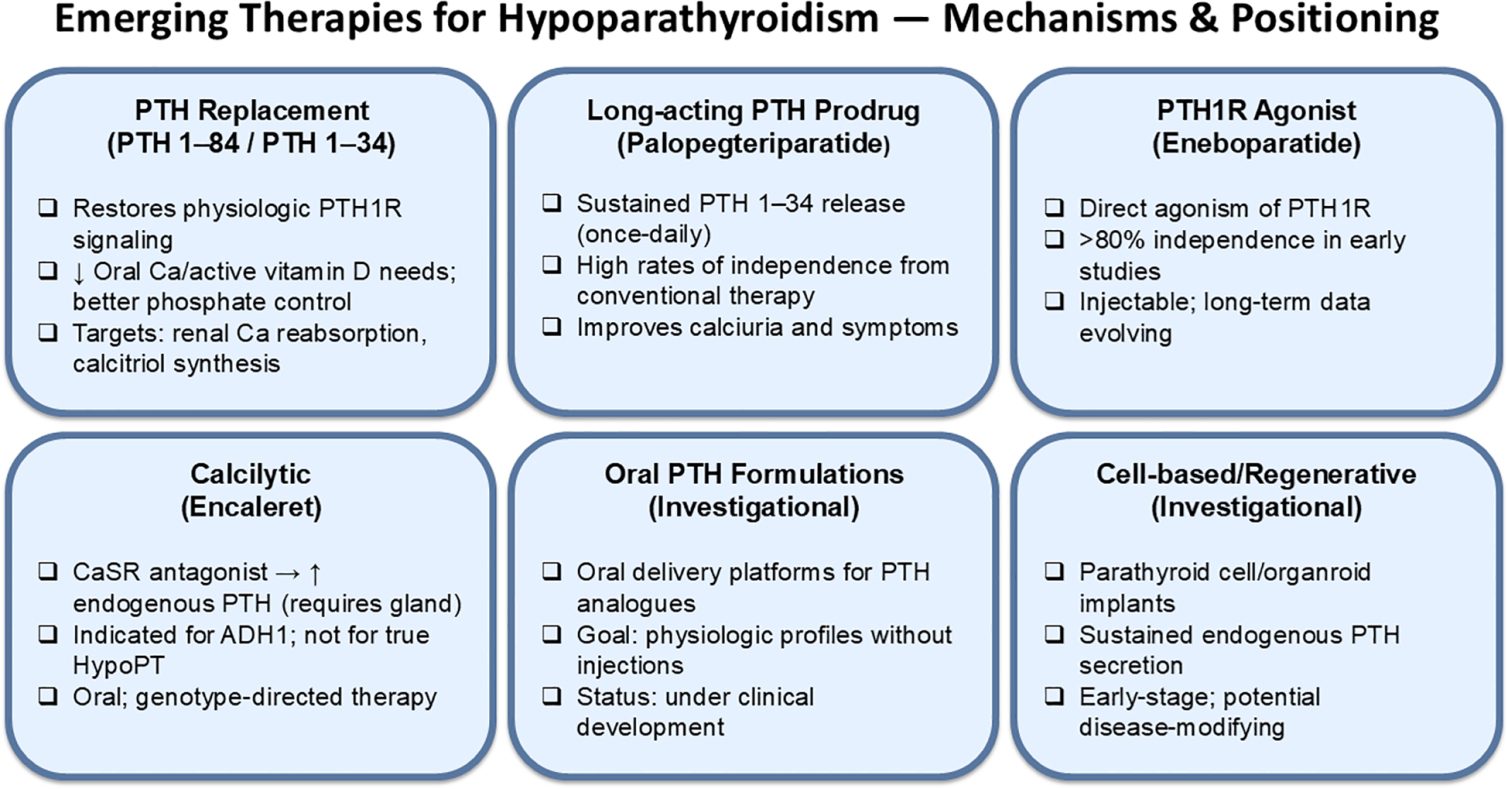
Emerging treatments for hypoparathyroidism with a brief description of their mechanism of action and key clinical data. PTH, parathyroid hormone; PTH1R, type 1 parathyroid hormone receptor; CaSR, calcium-sensing receptor; ADH1, autosomal dominant hypocalcemia type 1; HypoPT, hypoparathyroidism.

The potential advantages of these emerging therapies include a more physiologic regulation of calcium–phosphate balance, together with a markedly lower dependence on high doses of oral calcium and active vitamin D. A schematic representation of the emerging therapies for the treatment of hypoparathyroidism with a summary of their mechanisms of action and therapeutic positioning is shown in **Figure 3**. In many patients, these agents are associated with a reduction in hypercalciuria and offer improved symptomatic stability and quality of life, findings that have been consistently reported across clinical trials and real-world experience (35). At the same time, several limitations must be considered. Access and availability remain unequal between different regions and countries. Cost and logistical factors—including the need for injections, infusion devices, or closer biochemical monitoring—may also restrict broader implementation. Moreover, long-term safety data beyond the pivotal study windows are still emerging for some of these agents, underscoring the need for continued surveillance and extended follow-up in clinical practice (6).

### Clinical framing and present-day context

Chronic hypoparathyroidism is associated with complications that reflect sustained disturbances in calcium–phosphate balance rather than hypocalcemia alone. Skeletal effects include suppressed bone turnover, increased BMD at trabecular-rich sites, and alterations in microarchitecture; despite higher BMD, fracture risk remains uncertain and site-specific, with ongoing debate regarding vertebral versus non-vertebral patterns and how these may evolve under physiological PTH replacement and long-acting analogues (154, 155). Renal consequences—driven by the combination of high oral calcium/active vitamin D requirements and lack of PTH-mediated distal tubular calcium reabsorption—include hypercalciuria, nephrolithiasis, nephrocalcinosis, and, in some patients, progressive chronic kidney disease (156, 157). Beyond the kidney and skeleton, extra-skeletal calcifications are well recognized: intracranial (notably basal ganglia) calcifications with potential movement disorders, cognitive complaints, or neuropsychiatric symptoms; and, less commonly, ocular (cataracts) and soft-tissue/cardiovascular calcifications in the context of a chronically elevated calcium–phosphate product (156, 158).

These considerations frame modern management targets—biochemical stability with avoidance of hypercalciuria, preservation of renal function, and improved quality of life—and provide a pathophysiological rationale for PTH-based replacement strategies that can reduce supplement burden, lower urinary calcium, and potentially mitigate downstream complications. Conventional therapy can achieve stability in many, but hypercalciuria and renal sequelae remain concerns in a subset; PTH replacement and long-acting analogues are positioned to lower urinary calcium and reduce supplementation burden, with accumulating evidence of quality of life gains—all areas of ongoing longitudinal evaluation (35, 106).

In practice, calcium plus active vitamin D remains the first-line supplementation for many patients, whereas PTH replacement is directed to those with inadequate biochemical control, symptomatic fluctuations, hypercalciuria, renal complications, or reduced quality of life on conventional therapy. Modern classifications also distinguish transient from permanent postsurgical hypoparathyroidism by duration of persistence, with “permanent” typically defined by ongoing need for therapy beyond one year; this temporal framing clarifies prognosis and informs the decision to escalate from supplementation to physiological hormone replacement when appropriate (6, 106).

## Conclusions and future perspectives

The history of hypoparathyroidism spans from Owen’s publication in 1850 to the present day. Over the past 175 years, major discoveries and milestones have been achieved through the contributions of eminent figures in chemistry, biochemistry, molecular biology, histology, physiology, endocrinology, and clinical medicine (**Figure 1**). With regard to treatment of this hormonal deficiency, a century has elapsed since the discovery and clinical application of Collip’s extract, during which therapeutic options have evolved slowly but steadily. Over the past 25 years, however, the pace of progress has clearly accelerated, from the physiological and pediatric studies conducted by Winer to the approval of Natpara^®^ by U.S. and European regulatory authorities in the past decade. There has been growing interest in this disease and in pathophysiology-based treatments aimed at replacing the missing hormone. Clinical trials evaluating the efficacy and safety of PTH 1–34 and PTH 1–84, as well as prodrugs such as palopegteriparatide and PTH receptor agonists such as eneboparatide, have multiplied (159).

From the anatomical identification of the parathyroid glands to modern targeted therapies, historical breakthroughs have directly informed current clinical care. The correlation between scientific discoveries and current clinical practice and disease management can be summarized in five main points: (i) Diagnosis and classification now rest on harmonized biochemistry. The availability of accurate serum calcium and intact PTH assays enables timely diagnosis, distinction between transient and chronic postsurgical hypoparathyroidism, and structured longitudinal reassessment—critical for prognosis and treatment planning. (ii) Conventional therapy remains first-line but has intrinsic physiological limits. Calcium plus active vitamin D reliably corrects hypocalcemia by augmenting intestinal calcium absorption, yet does not restore renal PTH-dependent calcium reabsorption or phosphaturia, predisposing a subset of patients to hypercalciuria and renal sequelae despite biochemical control. (iii) Physiological replacement targets the mechanism. PTH-based therapies (full-length and long-acting analogues; PTH1R agonists) more closely re-establish normal renal handling of calcium and phosphate and support endogenous calcitriol generation, lowering supplement burden and often reducing urinary calcium—thereby aligning treatment with the underlying pathophysiology. (iv) Management should be complication-aware and outcome-oriented. Beyond symptomatic hypocalcemia, chronic disease reflects a broader disorder of mineral metabolism, with risks that include renal complications, site-specific skeletal effects under suppressed bone turnover, and extra-skeletal calcifications. Therapeutic targets should therefore integrate biochemical stability, urinary calcium reduction, renal preservation, and quality-of-life measures. (v) Innovation is reshaping the therapeutic horizon. Advances from receptor structural biology to next-generation drug design have accelerated options beyond daily injections—e.g., long-acting prodrugs, receptor agonists, and emerging platforms—signaling a shift toward durable, physiology-mimetic control of mineral homeostasis.

Taken together, these insights recast hypoparathyroidism as a systemic disorder of mineral regulation rather than hypocalcemia alone (159). Historical discovery has thus evolved into mechanism-based care: diagnose precisely, treat to restore physiology where appropriate, monitor for renal and skeletal safety, and pursue therapies that improve long-term outcomes and patient-reported well-being. Clinical research and innovative technological developments do not end here. Research efforts and progress achieved will undoubtedly transform both the clinical perception of this disease and the management of patients who suffer from it, as well as the results regarding its natural history and potential complications.
